# Social Force Model-Based Safety Evaluation of Intersections in Arterials Considering the Pedestrian Yield Rule

**DOI:** 10.3390/ijerph182312461

**Published:** 2021-11-26

**Authors:** Jiao Yao, Yuhang Li, Jiaping He

**Affiliations:** Business School, University of Shanghai for Science and Technology, Shanghai 200093, China; liyuhangemail@163.com (Y.L.); jiaping0219@126.com (J.H.)

**Keywords:** traffic engineering, social force model, safety evaluation, pedestrian yield rule, safety degree of pedestrian crossing, acceleration interference

## Abstract

To enhance the safety of pedestrians crossing the street, a series of new regulations regarding pedestrian yield has been proposed and widely implemented across cities. In this study, we first made some improvements to the social force model, in which pedestrian crossing at the intersection, drivers’ psychology of giving way, vehicle yield to pedestrians, vehicle yield in different directions, the influence of pedestrians crossing boundaries, and signal lamp groups on pedestrian behavior were considered. Furthermore, pedestrian crossing and vehicle yield safety models were established, based on which the comprehensive safety evaluation model of intersections in arterials was established, in which two indices—(1) the safety degree of pedestrian crossings and (2) vehicle acceleration interference—were combined with the entropy weight method. Finally, four types of intersections in arterials were studied using a simulation: the intersections between different levels of arterials, and intersections with one-time and two-times pedestrian crossings. Moreover, safety evaluation and analysis of those intersections, considering the rule of pedestrian yield, were conducted combined with the trajectory data from the VISSIM simulation. The relevant results showed that for pedestrians crossing the street, the pedestrian safety of two-time crossing is significantly higher than that of one-time crossing, and compared with the arterial, the pedestrian crossing distance of the sub-arterial is shorter, and the pedestrian perception is safer. Moreover, due to the herd psychology effect, the increase in pedestrian flow volume improves the safety perception of pedestrians at the intersection.

## 1. Introduction

Nowadays, with the concept of green transportation, greater attention has been given to the transit of pedestrians and bicycles. At intersections in urban areas, there are a large number of potential conflicts between pedestrians and vehicles; therefore, the safety of pedestrians crossing the street is a particularly important issue we should focus on.

Although the road traffic safety law of the People’s Republic of China [1] clearly defines the right of way of pedestrians, there are often conflict situations among traffic participants across different road grades, road facilities, and traffic conditions due to the complexity and diversity of traffic composition at intersections. In real-world scenarios, human–car conflict scenarios inevitably evolve into a mutual game that leads to a lack of safety of a relatively vulnerable group of traffic participants—pedestrians—on the street, which is a fatal traffic safety risk. In order to enhance the safety of pedestrians crossing the street, some regulations such as “Hangzhou Civilized Behavior Promotion Regulations” and “Shanghai Road Traffic Management Regulations” have been proposed and carried out in cities. To fulfill these regulations, vehicles must yield to pedestrians in case there are possible conflicts. Importantly, although the accident rate in China has reduced by 4% due to motor vehicles not considering the regulations, the number of accident deaths still accounts for 10% [2].

General intersection safety mainly considers the conflict in space but ignores the psychological perception of individuals. Therefore, combined with the social force model, which was modified by incorporating analyzing factors, we focus on the overall safety of the intersection. This paper uses the social force model to analyze the factors of intersection crossing, which is helpful for initiating reasonable improvements in urban intersections, and further promotes the safety of intersections.

The structure of this study is as follows. First, the social force model was modified by analyzing factors such as pedestrian crossing at intersections, driver psychology, the yielding of vehicles to pedestrian, the yielding of vehicles in different directions, the influence of crosswalk boundaries for pedestrian crossings, and the influence of signal lamp groups on pedestrian behavior. Second, the safety model of pedestrian crossing and the safety model of the vehicle yield was established. Third, with two indexes—(1) pedestrian crossing safety and (2) vehicle acceleration interference—the entropy weight method was used to evaluate the safety of arterial road intersections considering the pedestrian yield rule. Finally, with vehicle trajectory data in the VISSIM simulation platform, the safety evaluation and analysis of intersections in arteries considering the yield pedestrian rule were established.

## 2. Literature Review

Many studies that focus on urban intersection safety are based on historical accident statistics. For example, the Road Safety Manual, HSM [3], is a classic technical theoretical system in which the accident safety correction coefficient with different intersection types, traffic control methods, and other intersection design parameters are given in detail. Through the steps of the HSM, intersection safety evaluation can be efficiently carried out. Kang, B [4] evaluated the safety relevance of newly installed design elements at intersections in recent years by using the historical data of human–vehicle collisions from 2007 to 2015. The results showed that the size of pedestrian safety islands, the width of crosswalks and the width of lanes had a significant impact on the collision rate. Dixon, K et al. [5] evaluated the urban road conditions and compared them with historical accident data and road characteristics to identify and analyze the facility configuration, which may bring high safety risks. Ojo, T [6] evaluated pedestrians’ crossing behavior and safety at intersections with historical data and actual observational research, among which age, pedestrian condition, and zebra crossing location were shown to have a significant influence on safety.

Using traffic conflict technology to analyze the safety of intersections has also been a mainstream method in recent years. On the one hand, there are studies on the analysis and evaluation of various factors affecting the safety of participants at intersections, that quantify the severity of conflicts at intersections. For example, Porkhacheva, S. M [7] analyzed important factors affecting pedestrian crossing safety at the intersection, studied these factors, and proposed a method to evaluate the degree of danger of crosswalks at the intersection. Kumar, A et al. [8] determined the severity of conflict according to the demographics of pedestrians and their street-crossing behavior. They divided the conflict indicators into four severity levels by K-means clustering and used traffic conflict technology to explore the interaction between pedestrians and right-turn (left-bound) traffic at signalized intersections, subsequently evaluating the pedestrian safety at intersections. Shiomi, Y [9] used data from multiple regions and considered the lognormal obstacle model of regional and geometric attributes to quantify the factors affecting the traffic accident risk of various collision types. Yuan, L et al. [10] set a traffic safety evaluation model based on traffic conflict by using grey relevance theory and evaluated the safety level of intersections according to traffic channelization facilities and traffic historical conflict data of signalized intersections.

On the other hand, the evaluation was conducted by analyzing the conflicts between traffic participants. For example, Babu, S [11] studied the mixed traffic flow at unsignalized intersections and used the alternative PET index and passing speed of conflicting vehicles as evaluation indexes to evaluate the safety of intersections. Bai, L [12] evaluated the impact of traffic conflicts between electric bicycles and motor vehicles at signalized intersections on the safety of intersections. Zhang, H.L [13] used average vehicle delay, average vehicle travel time, time-to-collision (TTC), and post-encroachment time (PET) as evaluation indexes to study the impact of the speed limit at intersections on the safety and operation of intersections. Wang S.Q [14], based on the conflict between right-turn vehicles and pedestrians, established the collision safety evaluation model of pedestrians and right-turn vehicles at interchanges by using the three indexes of conflict time, post-invasion time, and safety reduction, and used the fuzzy C-means clustering algorithm. Guo Y.Y [15] analyzed the conflicts within intersections based on vehicle tracks and proposed a cross-section analysis and evaluation method of signalized intersections based on the Bayes traffic conflict model. Li, Q.Y and Sun, F [16] used the total number of conflicts and delays at intersections to analyze and evaluate the roundabout.

Among the existing intersection safety evaluation methods, the safety evaluation model based on historical accident statistics has obvious limitations, e.g., it is a post-accident evaluation method based on a large amount of historical accident data. Moreover, it is difficult to effectively apply this model in the prevention and control of intersection accidents. The intersection safety evaluation method based on traffic conflict technology can reflect the operation status of the actual traffic flow effectively and combined with the status of the operation of the intersection evaluation can be a good way to prevent the occurrence of traffic accidents. After summarizing the relevant research [7,8,9,10,11,12,13,14,15,16,17,18], it is found that the following four aspects can be further studied:

(1) Basic indicators cannot completely demonstrate the intersection operation safety considering pedestrian yield rules, such as TTC, PET, etc.
(2) When pedestrians and motor vehicles collide, the corresponding perception is completely different, which is rarely considered in mainstream intersection safety evaluation studies.
(3) The rule of yield to pedestrians is widely implemented, and the safety of pedestrians has been improved. The pedestrian characteristics have been changed compared with the traditional interaction between people and cars. On this basis, it is particularly important to consider the safety perception of pedestrians crossing the street.
(4) Vehicles yield to pedestrians to ensure the safety of pedestrians; however, the specific scene yield safety level is different. Pedestrians in the vehicle yield may still produce deceleration or acceleration behavior through the intersection; safety risks exist, so the intersection safety analysis in different scenarios must be further investigated.

Nowadays, there are abundant methods to analyze the crossing behavior of pedestrian intersections, and commonly used methods include cellular automata, the magnetic field force model, queuing theory, social force model, etc. [19,20,21,22]. The social force model was first proposed by D. Helbing. It is based on Newtonian mechanics and assumes that pedestrians move under the action of social forces. It is a well-suited social force model in the field of micro-pedestrian simulation and is widely used in pedestrian emergency evacuations. Some studies have also applied it to intersections. For example, LiY. X [23] proposed a modified social force model to simulate the through bicycle flow at mixed-traffic intersections. Anvari B [24] presented a three-layer micro-mathematical model which used the social force model to describe the behavior of pedestrians and vehicles in the layout of shared space and simulated the corresponding trajectory.

Under normal circumstances, pedestrian crossing has high flexibility. Pedestrian crossing behavior at different intersections will be different, and will be affected by common psychological behavior: for example, herd psychology and saving psychology—the former is also psychologically affected by the law and does not punish offenders’ thinking. In the latter, they save time by cutting corners and running red lights when they think they are safe. In the pedestrian crossing stage at the crosswalk, pedestrians are also affected by a series of social and environmental factors such as the pedestrian flow, traffic flow, and signal lights at the intersection; therefore, the social force model is also highly suitable for the pedestrian crossing safety analysis at the intersection.

In view of these problems, this study considers the pedestrian’s crossing behavior at intersections and proposes a safety evaluation method for intersections in arterial roads considering the pedestrian yield rule.

## 3. Methodology

Presently, many studies use the social force model to consider a greater number of factors in trajectory prediction [18,19,20,21,22,23,24], which enables a more accurate prediction. This paper proposes an intersection evaluation method based on the social force model.

This study considers the main factors affecting pedestrian safety during pedestrian crossings and combines these with the analysis of human–vehicle conflict. The comprehensive human-vehicle safety evaluation model for arterial road intersections considering the pedestrian yield rule based on the social force model is established. This can more accurately reflect the perceived safety of pedestrians crossing the street. In addition, the acceleration interference value is used to describe the yield safety at the intersection, and a comprehensive evaluation of yield safety at the intersection is achieved.

### 3.1. The Social Force Model of Intersections in Arterial Roads

Pedestrian crossing behavior at intersections is influenced by subjective thoughts and the external environment of intersections. At intersections in arterial roads, pedestrians must have a subjective willingness to cross the street; objectively, this is influenced by external factors such as traffic flow, signal lights, other pedestrians, crosswalk boundary, etc., resulting in appropriate crosswalk behavior. Motor vehicle drivers are similarly affected.

**Figure 1:**
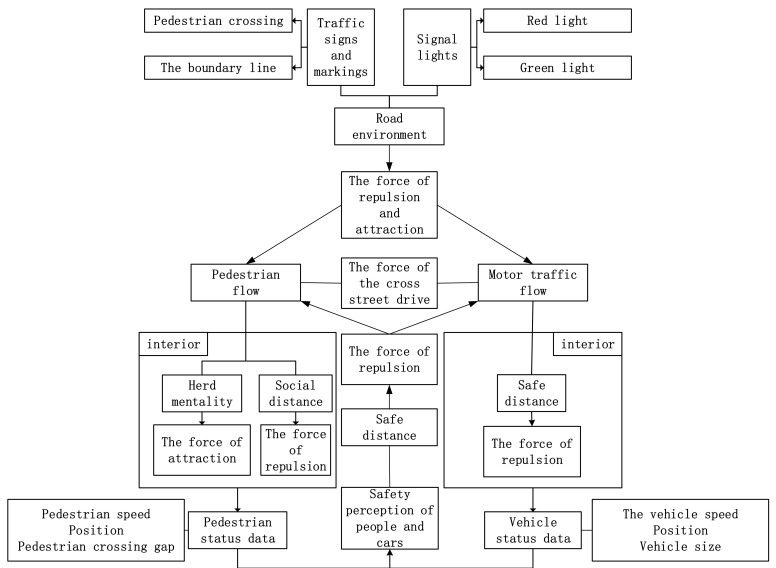
Interaction mechanism diagram of human, vehicle, and environment at intersection in the social force model.

The social force model can describe the core idea of interactions between individuals and between individuals and the environment in complex systems. In the complex system of an intersection, the mutual relationship between traffic participants and the environment is shown in Figure 1. Traffic participants interact and are affected by the environment in the intersection; the interaction force changes participants’ acceleration and velocity state. Different players have different safety awareness and corresponding behaviors crossing the street under the motion state changes.

Figure 1 analyzes the interaction mechanism between traffic participants and the environment at intersections based on the social force model. Participants at the intersection have the motivation to pass the intersection, and the social force model defines motivation as a driving force. Due to the distance between the participants, there is repulsive force and attraction; considering safety, the repulsive force exists between vehicles, people and cars, and people and people. In addition, there is a certain attraction brought by the crowd psychology among pedestrians; at the same time, traffic participants are affected by intersection environmental factors (signal lights, signs, and lines).

According to the interaction force of the social force model, this section is divided into four parts that study the self-driving force of pedestrians and vehicles, repulsion and attraction force between participants, the repulsive force of the crosswalk boundary, and the attraction, and repulsion force of the signal lamp group.

#### 3.1.1. Self-Driving Force of Pedestrians and Vehicles

Calculation of the self-driving force of pedestrians and vehicles at the intersection is reflected in the motivation of pedestrians and vehicles to pass the intersection at the expected speed, as shown in Equation (1):

(1) $${\vec{F}}_{\alpha }^{0}=m_{\alpha }\frac{1}{\tau_{\alpha }}\left(v_{\alpha }^{0}{\vec{e}}_{\alpha }-{\vec{v}}_{\alpha }\right)$$

where ${\vec{F}}_{\alpha }^{0}$ is the self-driving force of pedestrians and vehicles, $m_{\alpha }$ is mass of traffic participants α, $\tau_{\alpha }$ is the accelerated adaptation time required by participant α, $v_{\alpha }^{0}$ is the expected speed of traffic participant α during the action, expected speed is defined as the average driving speed of participants when they pass the intersection without interference from the surrounding road users. ${\vec{e}}_{\alpha }$ is the unit vector of the velocity direction of traffic participant α, and ${\vec{v}}_{\alpha }$ is the actual velocity vector of traffic participant α.

#### 3.1.2. Basic Model of Participant Repulsive Force

Repulsive force refers to the force to maintain a certain safe distance between traffic participants (car to car, person to person, person to vehicle) in the social force model at the intersection: when the social field is regarded as a circle, the repulsive force can be expressed as shown in Equation (2):

(2) $${\vec{F}}_{\alpha \beta }^{soc}=A_{\alpha }\exp\left[\frac{\left(r_{\alpha \beta }-d_{\alpha \beta }\right)}{B_{\alpha }}\right]{\vec{n}}_{\alpha \beta }$$

where ${\vec{F}}_{\alpha \beta }^{soc}$ is the social force of participant β to participant α, $A_{\alpha }$ is the strength coefficient between estimated participant α and other participants, $r_{\alpha \beta }$ is the sum of radii of participant α and participant β; $d_{\alpha \beta }$ is the distance between participant α and participant β; $B_{\alpha }$ is the force strength coefficient of participant α; and ${\vec{n}}_{\alpha \beta }$ is the unit vector of participant β pointing to participant α.

In reality, since traffic participants all have forward speed, the social field is more consistent with the elliptical social field, as shown in Equation (3):

(3) $${\vec{F}}_{\alpha \beta }^{soc}=A_{\alpha }\exp\left[\frac{\left(-b_{\beta }\right)}{B_{\alpha }}\right]{\vec{n}}_{\alpha \beta }$$

(4) $$b_{\beta }=\frac{\sqrt{\left(∥{\vec{P}}_{\beta }-{\vec{P}}_{\alpha }∥+∥{\vec{P}}_{\beta }-{\vec{P}}_{\alpha }+v_{\beta }\Delta t{\vec{e}}_{\beta }∥\right)-{\left(v_{\beta }\Delta t\right)}^{2}}}{2}$$

where $b_{\beta }$ is the radius length of the social field generated by participant β. ${\vec{P}}_{\beta }$ is the position vector of participant β, and ${\vec{P}}_{\alpha }$ is the position vector of participant α.

**Figure 2:**
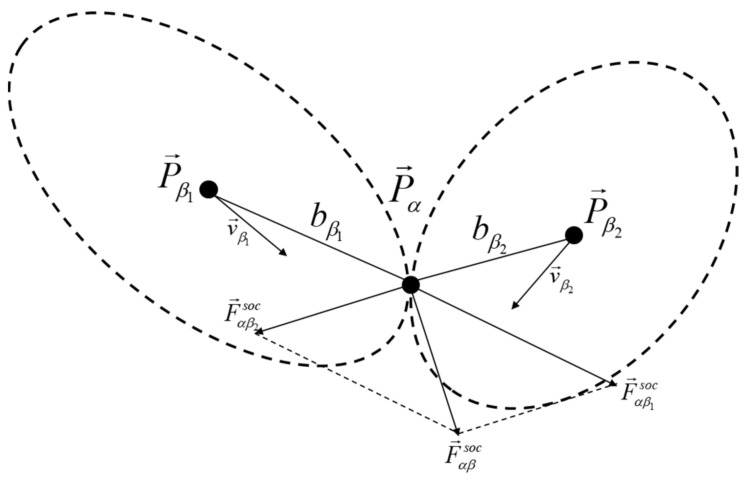
Diagram of social force calculation for multiple participants.

When multiple participants generate social forces, take two participants as an example. Participants $\beta_{1}$ and $\beta_{2}$ act on participant α, and each participant will generate an elliptical social field, which generates a repulsive force on participant α. The resultant force can be calculated by their respective repulsive force on α, as shown in Figure 2.

#### 3.1.3. The Repulsion and Attraction Force among Pedestrians

In order to maintain certain distance among pedestrians, the social force mainly consists of two parts, namely, pedestrian repulsive force and pedestrian attraction force.

**(1) Pedestrian repulsive force.**

**Figure 3:**
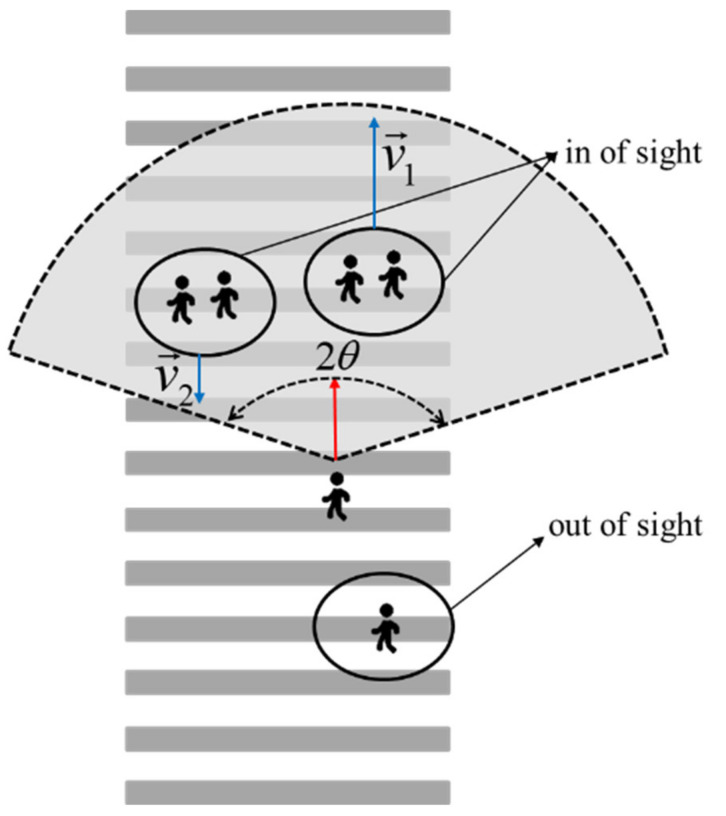
Schematic of pedestrian visibility range.

Considering the visual impact of pedestrians, that is, the impact strength of pedestrians in and out of sight is different, as shown in Figure 3:

Compared with pedestrians in sight, pedestrians out of sight have a weak repulsive effect on target pedestrians. Assuming that the effective visual angle of people is 2*θ*, the direction weight is introduced, as shown in Equation (5):

(5) $$\omega_{\alpha \beta }^{}=\left\{\begin{array}{cc} 1, & \vec{e}{\vec{F}}_{\alpha \beta }^{pex}\geq \cos\theta ∥{\vec{F}}_{\alpha \beta }^{pex}∥\\ c, & otherwise \end{array}\right.$$

where *c* is a constant in the range [0–1].

Then, the social repulsive force of pedestrians can be expressed as:

(6) $${}^{i}{\vec{F}}_{p}^{pex}={}^{i-}{\vec{F}}_{p}^{pex}+{}^{i+}{\vec{F}}_{p}^{pex}$$

(7) $${}^{i-}{\vec{F}}_{p}^{pex}=\sum \omega_{\alpha \beta_{j}}{\vec{F}}_{\alpha \beta_{j}}^{soc}$$

(8) $${}^{i+}{\vec{F}}_{p}^{pex}=\sum {\vec{F}}_{\alpha \beta_{j}}^{soc}$$

where ${}^{i}{\vec{F}}_{p}^{pex}$ is the total pedestrian social repulsive force of pedestrians *I*, ${}^{i-}{\vec{F}}_{p}^{pex}$ is the social force of pedestrians outside the range of visibility of pedestrian *i*, and ${}^{i+}{\vec{F}}_{p}^{pex}$ is the social force of pedestrians within the range of visibility of pedestrian *i.*

**(2) Pedestrian attraction.**

Pedestrians crossing the street often have herd psychology. Considering the conformity factor and combining with the social force model, there is a certain attraction among pedestrians crossing the street, called conformity force, which can be expressed by Equations (9) and (10):

(9) $${\vec{F}}_{\alpha \beta }^{patt}=A_{p}{\vec{n}}_{\alpha \beta }f\left(d_{\left(\alpha -\beta \right)},d\right)$$

(10) $$f\left(d_{\left(\alpha -\beta \right)},d\right)=\left\{\begin{array}{cc} 0 & d_{\left(\alpha -\beta \right)}\leq d\\ 1 & d_{\left(\alpha -\beta \right)}>d \end{array}\right.$$

where ${\vec{F}}_{\alpha \beta }^{patt}$ is the attraction among pedestrians, $A_{p}$ is the pedestrian attraction strength parameters, $f\left(d_{\left(\alpha -\beta \right)},d\right)$ is a piecewise function, $d_{\left(\alpha -\beta \right)}$ is the distance between pedestrians and pedestrian group; d is the distance threshold, the calculation formula of the threshold is $\frac{N-1}{2}$ [25], and N is the number of people in the pedestrian group.

#### 3.1.4. Repulsive Force of the Crosswalk Boundary

**Figure 4:**
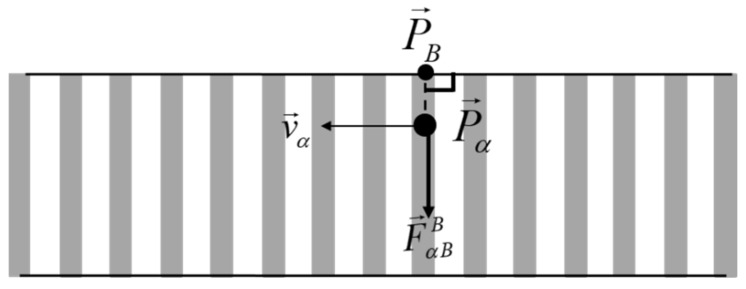
Schematic of boundary force of pedestrian crossing.

According to the Road Traffic Safety Law of the People’s Republic of China, when pedestrians pass through intersections or cross roads, they shall take pedestrian crossings or street crossing facilities; therefore, this study assumes that the pedestrian crossing is completed in the crosswalk, without considering the illegal behaviors that are absent from the crosswalk. Therefore, the crosswalk boundary exerts a repulsive force on the pedestrian, forcing the pedestrian not to leave the crosswalk area, as shown in Figure 4.

In this case, the boundary force of the pedestrian crossing can be expressed by ${\vec{F}}_{\alpha B}^{B}$, calculated by Equation (11).

(11) $${\vec{F}}_{\alpha B}^{B}=A_{\alpha B}\exp\left(-B_{\alpha }∥{\vec{P}}_{B}-{\vec{P}}_{\alpha }∥\right){\vec{n}}_{\alpha B}$$

where ${\vec{F}}_{\alpha B}^{B}$ is the boundary forces on pedestrians, $A_{\alpha B}$ is the strength coefficient between the crosswalk boundary and pedestrians, $B_{\alpha }$ is the force range coefficient; ${\vec{P}}_{B}$ is the boundary point of pedestrian crossing near the pedestrian, and ${\vec{P}}_{\alpha }$ is the position with the pedestrian; ${\vec{n}}_{\alpha B}$ is the unit vector whose boundary points to pedestrian α.

#### 3.1.5. Repulsion and Attraction of the Signal Lamp Group to Pedestrians

It is postulated that when the signal lamp group is green, all pedestrians choose to cross the street, and when the signal light is red, pedestrians choose to stop entering the crosswalk. In addition, the green flashing is a special signal stage, which is considered to be the end of the time for pedestrians to safely cross the street at the crosswalk. According to relevant studies [26], pedestrian speed is significantly higher during the flashing green light than during the non-flashing green light stage. Therefore, this study considers that during the red light period, pedestrians are subjected to the repulsive force of the signal light, while pedestrians entering during the green light flashing will have a greater crossing speed. That is, when the signal light flashes green (or during the countdown), it produces an attraction to pedestrians.

**(1) Red light repulsive force.**

The red light repulsive force on pedestrians can be calculated by the related social repulsive force model, as shown in Equation (12).

(12) $${\vec{F}}_{\alpha D}^{exc}=A_{D}\exp\left[\frac{\left(-b_{D}\right)}{B_{D}}\right]{\vec{n}}_{\alpha D}$$

where ${\vec{F}}_{\alpha D}^{exc}$ is the social repulsive force of the red light to pedestrians, $A_{D}$ is the repulsion strength of the signal lamp, $B_{D}$ is the range parameter, $b_{D}$ is the distance between pedestrians and the signal lamp.

**(2) Green light attraction.**

**Figure 5:**
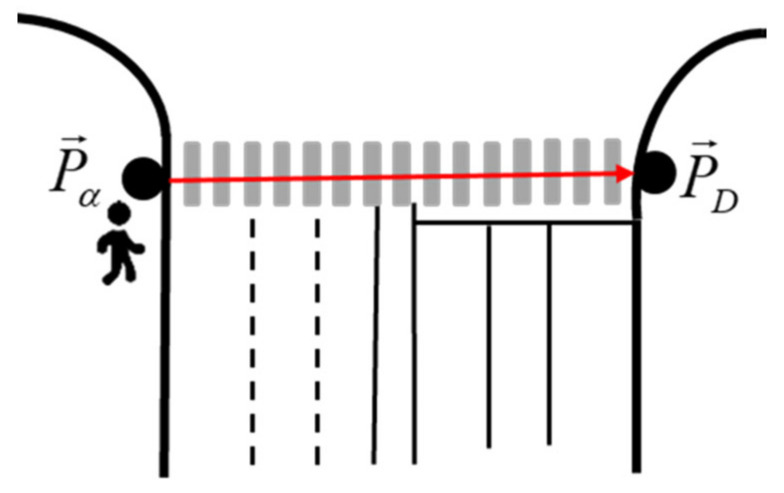
Schematic of pedestrian crossing position.

During the green light, it is assumed that the pedestrian will move towards the target exit position before crossing the street, as shown in Figure 5.

It is assumed that the attraction of the green light to pedestrians is linearly related to the current distance to the desired exit position. The farther away the walker is from the desired exit, the stronger the attraction. Therefore, the attraction of the green light to pedestrians can be expressed as Equation (13).

(13) $${\vec{F}}_{\alpha D}^{att}=\left\{\begin{array}{c} 0\\ \left(-A_{D}^{att}∥{\vec{P}}_{\alpha }-{\vec{P}}_{D}∥+B_{{}_{D}}^{att}\right){\vec{n}}_{\alpha D} \end{array}\right.\begin{array}{c} \mathrm{during}\;\mathrm{green}\;\mathrm{but}\;\mathrm{not}\;\mathrm{during}\;\mathrm{flashing}\;\mathrm{green}\\ \mathrm{flashing}\;\mathrm{green} \end{array}$$

where ${\vec{F}}_{\alpha D}^{att}$ is the social attraction of the green light to pedestrians, $A_{D}^{att}$ is the attraction strength of the signal lamp, $B_{{}_{D}}^{att}$ is the range parameter of the signal lamp, ${\vec{P}}_{D}$ is the position vector of the pedestrian leaving the road.

In summary, the social attraction and repulsion of the signal lamp group to the pedestrian can be expressed as follows:

(14) $${\vec{F}}_{\alpha D}^{soc}=\left\{\begin{array}{c} {\vec{F}}_{\alpha D}^{exc}\\ {\vec{F}}_{\alpha D}^{att} \end{array}\right.\begin{array}{c} \;\mathrm{red}\;\mathrm{light}\;\mathrm{stage}\\ \;\mathrm{green}\;\mathrm{light}\;\mathrm{stage} \end{array}$$

#### 3.1.6. Model Summary

According to the above analysis, at the intersection, The main social forces for pedestrians across the street are ${\vec{F}}_{p}^{isoc}$, the force of the *i*th pedestrian is composed of five parts, namely, the self-driving force of the pedestrian F→ip0, the repulsive force on the pedestrian by the pedestrian and motor vehicle participants ${}^{i}{\vec{F}}_{p}^{soc}$, the attraction force among pedestrians ${}^{i}{\vec{F}}_{p\beta }^{patt}$, the boundary repulsive force of the crosswalk ${}^{i}{\vec{F}}_{pB}^{B}$, and the repulsive force ${}^{i}{\vec{F}}_{\alpha D}^{soc}$ caused by the signal lamp, as shown in Equation (15).

(15) $${\vec{F}}_{p}^{isoc}={}^{i}{\vec{F}}_{p}^{0}{+}^{i}{\vec{F}}_{p}^{soc}+{}^{i}{\vec{F}}_{p\beta }^{patt}+{}^{i}{\vec{F}}_{pB}^{B}+{}^{i}{\vec{F}}_{\alpha D}^{soc}$$

where ${}^{i}{\vec{F}}_{p}^{soc}$ is the social repulsive force of pedestrians by other participants.

The social force on the vehicles at the crosswalk at the intersection is ${\vec{F}}_{v}^{isoc}$, which is mainly composed of vehicle self-driving force F→iv0 and repulsive force F→ivsoc of other participants, as shown in Equation (16).

(16) F→visoc=F→iv0+F→ivsoc

### 3.2. Safety Evaluation Model of Intersection Considering Yield Pedestrian Rule

According to the above analysis, vehicles and pedestrians are subjected to social forces ${\vec{F}}_{v}^{isoc}$ and ${\vec{F}}_{p}^{isoc}$ respectively, and their impact at the intersection can be regarded as the impact on the psychological perceived safety of pedestrians and vehicles crossing the street. Based on the above social force model, this section uses two indicators: (1) safety degree of pedestrian crossing and (2) vehicle acceleration interference value to evaluate the crossing safety of pedestrians and vehicles, respectively, and integrates both through the entropy weight method to conduct a comprehensive safety evaluation of arterial road intersections considering the rule of yield to pedestrians.

#### 3.2.1. Pedestrian crossing Safety Model

**Figure 6:**
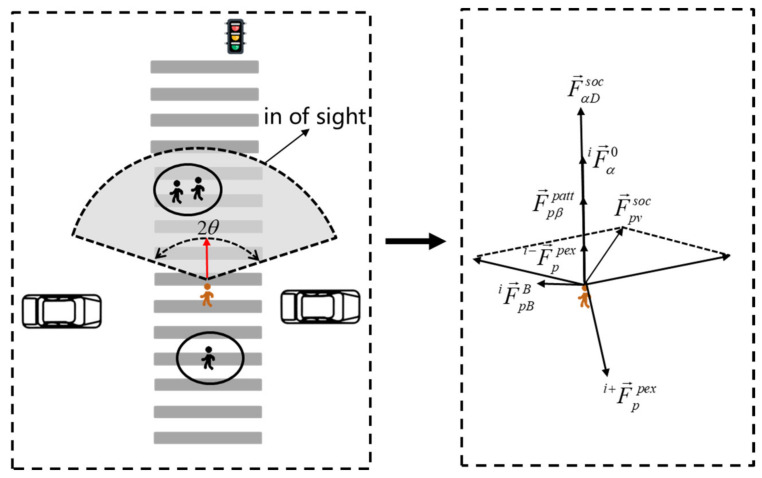
Pedestrian force analysis.

Based on analysis of the social force model of Section 1, the force of the pedestrian crossing process is modeled. The force of the *i*th pedestrian is composed of four parts and seven social forces, including pedestrian self-driving force, crosswalk boundary force, social force of other participants on pedestrians (including vehicle, pedestrian in sight, pedestrian out of sight, and conformity force), attraction, and repulsion force of signal lights, as shown in Equation (17) and Figure 6.

(17) $${\vec{F}}_{p}^{isoc}={}^{i}{\vec{F}}_{p}^{0}+{}^{i}{\vec{F}}_{pB}^{B}+{}^{i}{\vec{F}}_{pv}^{soc}+{}^{i-}{\vec{F}}_{p}^{pex}+{}^{i+}{\vec{F}}_{p}^{pex}+{\vec{F}}_{p\beta }^{patt}+{\vec{F}}_{\alpha D}^{soc}$$

The above model is static and does not consider the movement of pedestrians. In reality, it is necessary to conduct modeling and analysis on the continuous stage of the entire pedestrian crossing behavior. Therefore, the pedestrian crossing time $t_{i}$ should be taken into account in the average social force of pedestrian crossing at the arterial road intersection. The average social force of pedestrian crossing can be expressed in Equation (18):

(18) $$\bar{F_{p}^{isoc}}=\frac{\int_{0}^{t_{i}}{\vec{F}}_{p}^{isoc}(t)dt}{t_{i}}$$

where $\bar{F_{p}^{isoc}}$ is the average social force of the pedestrian.

In the process of crossing the street, pedestrians are affected by the social forces induced by the intersection traffic flow and traffic environment. Pedestrians crossing the street will perceive the intersection situation and their psychological and physiological behavior will be affected based on the perceived situation. This mapping can be reflected in the psychological and physical tension of individuals, referred to as people’s panic behavior. Based on panic behavior, the safety index of the safety degree of pedestrian crossing is defined. The greater the social force on pedestrians, the more panic they feel and the less they perceive safety; the closer the perceived safety is to 1, the safer the pedestrian crossing perception is; the closer it is to 0, the worse the safety of crossing the street at the intersection is. The perceived safety of crossing the street is defined as the gap between the perceived safety and the force experienced by the accident, as shown in Equation (19). The greater the value, the safer the pedestrian crossing the street.

(19) $$ps=|1-\frac{\bar{F_{i}}}{F_{lim}}|$$

here, ps is the safety degree of pedestrian crossing, $\bar{F_{i}}$ is the average psychological bearing social force of pedestrians *i*, $F_{lim}$ is the social force that pedestrians bear psychologically in the extreme situation (maximum force) of an accident.

For calculating the social force in extreme cases, the model mainly considers the social force of pedestrians in the case of a collision, as shown in Equation (20):

(20) $$F_{lim}=\underset{P_{c}\to P_{i}}{\lim}({\vec{F}}_{p}^{isoc})$$

where $P_{c}\to P_{i}$ is the position where the vehicle approaches the pedestrian *i.*

The closer the safety degree of pedestrian crossing ps is to 0, the more unsafe the pedestrian crossing is. In other words, the greater the influence of social forces is, the more dangerous pedestrians perceive it.

#### 3.2.2. Vehicle Yield Safety Model

Similar to the force of pedestrians crossing the street at an intersection, the social force of the vehicle is also composed of four parts, including the driving force of the vehicle, social force caused by the vehicle in front of the vehicle, social force caused by the vehicle behind the vehicle, and social force caused by the pedestrian in sight of the vehicle, as shown in Equation (21):

(21) F→vi=F→iv0+F→i−vsoc+F→i+vsoc+F→vpsoc

where ${\vec{F}}_{v}^{i}$ is the social force on vehicle *I*, F→iv0 is the self-driving force of the vehicle; ${}^{i-}{\vec{F}}_{v}^{soc}$, ${}^{i+}{\vec{F}}_{v}^{soc}$ is the social force of the rear and front vehicles on the current vehicle, respectively, ${\vec{F}}_{vp}^{soc}$ is the social force of pedestrians on vehicles within the range of visibility.

Three kinds of social forces, namely, the self-driving force of the vehicle, social force caused by the vehicle in front, and social force caused by the vehicle behind, were described in Part 1. The social force of pedestrians in the range of visibility to the vehicle can be expressed as Equation (22):

(22) $${}^{i}{\vec{F}}_{vp}^{soc}={}^{i+}{\vec{F}}_{vp}^{soc}+{}^{i-}{\vec{F}}_{vp}^{soc}$$

where ${}^{i+}{\vec{F}}_{vp}^{soc}$, ${}^{i-}{\vec{F}}_{vp}^{soc}$ is the social force of the left and right pedestrians on the vehicle.

When a vehicle conflicts with pedestrians, it will have a certain social force impact on the pedestrians around the vehicle. Based on the gap between vehicles crossing, this chapter proposes the evaluation section of vehicle yielding safety, then uses the vehicle’s social field to calculate the corresponding social force and carries out a modeling analysis on the continuous stage in the evaluation section of vehicle yielding safety.

**Figure 7:**
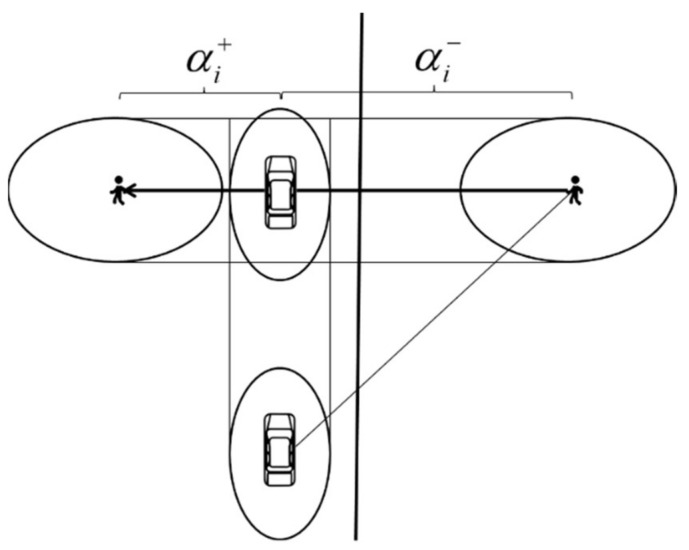
Pedestrian gap time interval diagram.

The method for determining the safety evaluation section of vehicle yield is as follows, based on the center line of the vehicle and the pedestrian line, the pedestrian gap time distance is divided into two parts, front pedestrian gap time interval $\alpha_{i}^{+}$ and rear pedestrian gap time interval $\alpha_{i}^{-}$, as shown in Equation (23) and Figure 7:

(23) $$\alpha_{i}=\alpha_{i}^{+}+\alpha_{i}^{-}$$

where $\alpha_{i}$ is the pedestrian gap time interval. $\alpha_{i}^{+}$ is the front pedestrian gap time interval, $\alpha_{i}^{-}$ is the rear pedestrian gap time interval.

**Figure 8:**
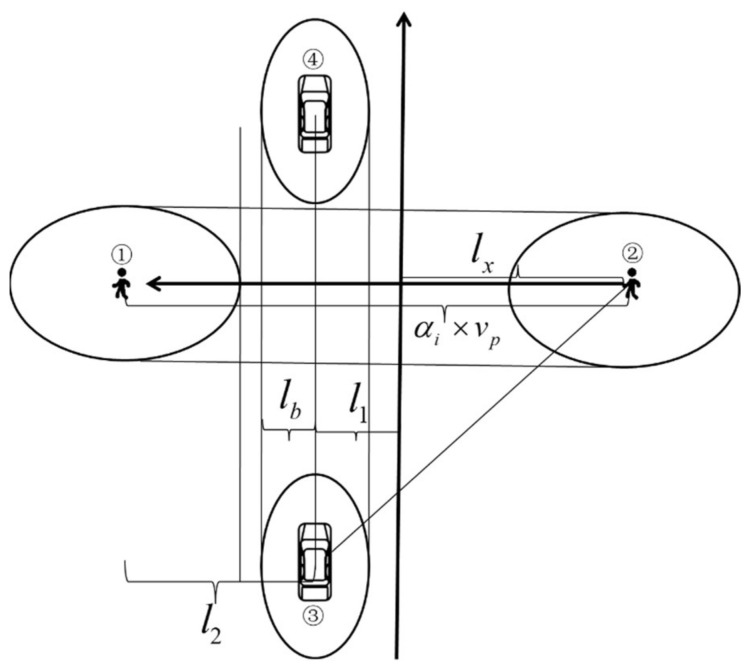
Pedestrian and vehicle position map.

The coordinate axes are constructed based on the pedestrian crossing line at the intersection crosswalk and the road boundary line, as shown in Figure 8. According to the motion coordinates, the distance between people and vehicles that varies with time can be calculated, and based on this, the social force of pedestrians to vehicles can be calculated.

In Figure 8 $l_{2}$ is the horizontal distance between people and cars; $l_{b}$ is the length of the short half axis of the depot; $l_{1}$ is the distance between the vehicle and the road boundary line; $l_{x}$ is the distance between the pedestrian and the road boundary when the vehicle can observe the pedestrian. The coordinate axis is constructed based on the pedestrian route and the road boundary. Then, the position coordinate points of both the pedestrian and the vehicle can be calculated:

In Figure 8, Position ➀ can be represented as $\left(l_{1}+l_{2},0\right)$, Position ➁ can be represented as $\left(-l_{x},0\right)$, Position ➂ can be represented as $\left(l_{1},-\frac{l_{x}+l_{1}}{\tan\theta }\right)$, Position ➃ can be represented as $\left(l_{1},\frac{w_{c}+2l_{s}}{2}\right)$; where: 2θ is the perspective range of people and $w_{c}$ is the width of the pedestrian crossing.

When the pedestrian gap time interval can meet the requirements of vehicles passing through, calculate the social force of vehicles on pedestrians in the crosswalk of the intersection during the whole crossing process, calculate the distance between people and vehicles based on the coordinate points, and sum it with the basic social force model to obtain the social force of vehicles on pedestrians, as shown in Equation (24):

(24) $${\vec{F}}_{pv}^{soc}=\int_{0}^{\alpha_{i}}A_{p}\exp\left[\frac{\left(-b_{pv}\right)}{B_{p}}\right]{\vec{n}}_{pv}$$

where ${\vec{F}}_{pv}^{soc}$ is the social force of vehicles to pedestrians.

When the pedestrian gap time interval $\alpha_{i}$ is too small, the vehicle needs to yield and wait for the next passable gap, then, the social force of the two pedestrian gaps is calculated for the vehicle. The force of the vehicle affected by the pedestrian during the yield period can be expressed by Equation (25):

(25) $${}^{\pm }{\vec{F}}_{vp}^{soc}=\int_{0}^{\alpha_{i}}A_{v}exp\left[\frac{\left(-b_{vp}\right)}{B_{v}}\right]{\vec{n}}_{vp}$$

where ${}^{\pm }{\vec{F}}_{vp}^{soc}$ is the social force of front and rear pedestrians to vehicles.

In conclusion, the average social force received by vehicle I during the entire safety evaluation period can be expressed by Equation (26):

(26) $$\bar{F_{v}^{isoc}}=\frac{\sum_{i=0}^{n}\int_{0}^{\alpha_{i}}{\vec{F}}_{v}^{isoc}(t)dt}{\sum_{i=0}^{n}\alpha_{i}}$$

where ${\vec{F}}_{v}^{isoc}$ is the total social force on the vehicle and *n* is the number of pedestrian gaps for the vehicle to yield.

When the social force of the vehicle is unbalanced, the vehicle needs to accelerate or slow down to avoid conflict. Herman proposes the concept of acceleration interference [27], and studies the relationship between acceleration interference and safety and comfort, pointing out that it is more accurate to evaluate safety with acceleration interference. Based on the acceleration interference value, the intersection passing safety evaluation value model is constructed. The mathematical formula of acceleration interference is expressed by Equation (27):

(27) $$I=\sqrt{\frac{1}{T}\int_{0}^{T}{\left[a(t_{i})-\bar{a}\right]}^{2}dt}$$

where I is the acceleration interference, T is the analysis acceleration interference time period, and a is the acceleration.

In combination with the social force on the vehicle, the average acceleration interference value of the vehicle passing through the intersection can be calculated through Equations (28) and (29). The greater the average acceleration interference value, the less stable the vehicle driving and the lower the safety.

(28) $$I_{i}=\sqrt{\frac{1}{\alpha_{i}}\int_{0}^{\alpha_{i}}{\left[\frac{∥{\vec{F}}_{v}^{isoc}∥-∥\bar{F_{v}^{isoc}}∥}{m}\right]}^{2}dt}$$

(29) $$\bar{I}=\frac{\sum_{1}^{N}I_{i}}{N}$$

where, $\bar{I}$ is the average acceleration interference value, N is the number of vehicles.

#### 3.2.3. Comprehensive Safety Evaluation Model of Pedestrian and Vehicle

Based on the pedestrian and vehicle safety model in Section 2, in order to avoid the interference of subjective factors, the weight of pedestrian crossing safety and vehicle acceleration interference index is objectively and accurately determined, and the entropy weight method is used to set the comprehensive safety evaluation model of both the pedestrian and the vehicle at the intersection [28]. The specific steps are as follows,

➀ For n indicators, take m sample values, and $x_{ij}$ is the value of the *j* indicator of the ith sample.

➁ Standardization of indicators.

Positive indicator: standardization of pedestrian safety, as shown in Equation (30):

(30) $$x^{\prime }{}_{ij}=\frac{x_{ij}-\min\left\{x_{1j},\ldots ,x_{nj}\right\}}{\max\left\{x_{1j},\ldots ,x_{nj}\right\}-\min\left\{x_{1j},\ldots ,x_{nj}\right\}}$$

Negative indicator: standardization of vehicle acceleration interference value, as shown in Equation (31):

(31) $$x^{\prime }{}_{ij}=\frac{\min\left\{x_{1j},\ldots ,x_{nj}\right\}-x_{ij}}{\max\left\{x_{1j},\ldots ,x_{nj}\right\}-\min\left\{x_{1j},\ldots ,x_{nj}\right\}}$$

➂ Calculate the entropy value corresponding to each indicator, as shown in Equations (32) and (33).

(32) $$E_{j}=-In{\left(n\right)}^{-1}\sum_{i=1}^{n}p_{ij}Inp_{ij}$$

(33) $$p_{ij}=x^{\prime }{}_{ij}/\sum_{i=1}^{n}x^{\prime }{}_{ij}$$

where $E_{j}$ is the information entropy and $p_{ij}$ is the proportion after sample standardization.

➃ Calculate the weight of each index, as shown in Equation (34):

(34) $$\omega_{j}=\frac{1-E_{j}}{n-\sum E_{j}}$$

where $\omega_{j}$ is the weight of index.

➄ Calculate the comprehensive safety evaluation index of intersection considering yield rules, as shown in Equation (35):

(35) $$Ys=\sum_{j=1}^{m}\omega_{j}x^{\prime }{}_{ij}$$

where Ys is the comprehensive safety evaluation index; *m* takes 2, the first index is safety degree of pedestrian crossing, and the second index is acceleration interference value.

## 4. Case Studies

### 4.1. Data Investigation

**Figure 9:**
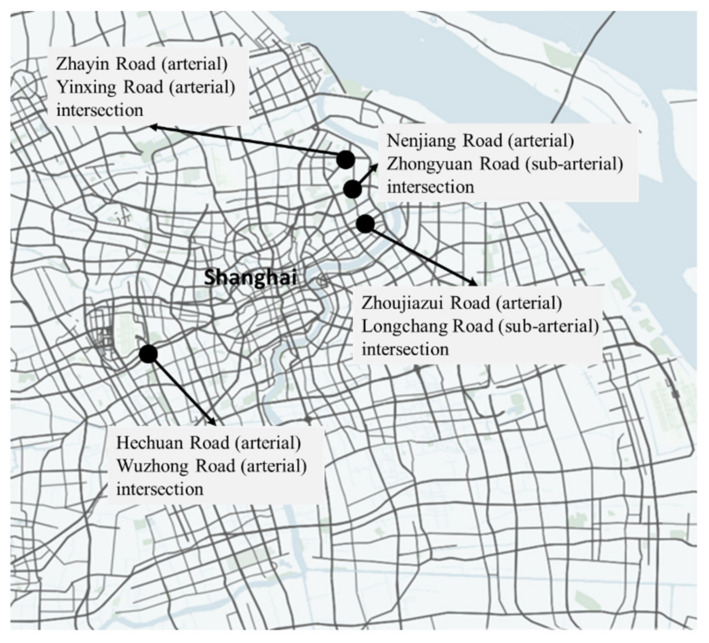
Location distribution of intersections in arterial in case study.

In order to evaluate the safety level of multiple types of arterial road intersections considering the pedestrians yield rule, this paper selects four arterial road intersections in the 13th five-year plan of Shanghai for relevant data collection [29], trajectory simulation, model calculation, and result analysis, as shown in Table 1. The specific location of the case intersection is shown in Figure 9. It is mainly distributed in densely populated areas at the junction of urban and rural areas. These arterial road intersections include intersections between arterial and arterial, intersections between arterial and sub-arterial, as well as intersections with 1- and 2-time crossings of pedestrians, which basically covers all types of arterial road intersections. Related data collection was carried out on weekday evening peak periods (16:45–17:45) with good weather at four intersections for two weeks and ten days.

The traffic simulation software Vissim is used to simulate the traffic conditions of real-world intersections. Inputs include: basic geometric contour, proportion of vehicles in each direction of traffic flow, signal timing, and other parameters obtained from the investigation into the Vissim model, and the corresponding trajectory data of simulated pedestrians and vehicles is output. Furthermore, the trajectory data is imported into the surrogate safety assessment model (SSAM) for safety evaluation. Concurrently, the same trajectory data is used in combination with the comprehensive evaluation model of the pedestrian yield rule for safety evaluation.

### 4.2. Model Parameter Calibration

(1) Calibration value of Vissim simulation parameters.

According to the investigation and related literature review [30,31,32], the values of some Vissim simulation parameters are shown in Table 2:

(2) Parameter calibration of the social force and safety evaluation model.

The parameters of the social force model are divided into measurable parameters and non-measurable parameters. According to the investigation and relevant literature [27,33,34,35], the values of relevant measurable parameters in the social force model are shown in Table 3.

Non-measurable parameters are estimated by using the maximum likelihood estimation method [36]. Assuming that the resultant social force follows a normal distribution with mean value μ and variance σ, the corresponding maximum likelihood function is:

(36) $$L\left(a_{i}^{j}\left(\theta \right)\left|\mu ,\sigma \right.\right)=\prod \frac{1}{\sigma \sqrt{2\pi }}\exp\left(\frac{-{\left(a_{i}^{j}\left(\theta \right)-\mu \right)}^{2}}{2\sigma^{2}}\right)=\frac{{\left(2\pi \right)}^{-\frac{n}{2}}}{\sigma^{2}}\exp\left(\frac{-\sum {\left(a_{i}^{j}\left(\theta \right)-\mu \right)}^{2}}{2\sigma^{2}}\right)$$

where $a_{i}^{j}\left(\theta \right)$ represents the acceleration of participant *j* at time *i*, θ is the parameter vector.

Take the derivative of the maximum likelihood function with respect to the parameter vector θ, let the derivative be 0, and the maximum value of the maximum likelihood function can be calculated. At this time, the component value of the parameter vector θ is the result of parameter calibration. The parameter estimation results are displayed in Table 4.

In order to determine the safety impact of vehicle yield to pedestrians on the intersection of arterial roads under different pedestrian flow volume scenarios, according to ‘Code for Design of Urban Road Engineering’, on the premise that the upper limit of a single zebra crossing is 1580 people per hour, and the maximum pedestrian flow ratio in the zebra crossing at the existing intersection is 0.33. The value range of pedestrians at the intersection is determined as [200–4700], which is divided into 20 sections. The interval step is 225 and is then divided into each direction according to the proportion of existing pedestrian flow volume.

According to different pedestrian flow volumes, the trajectory simulation of the intersection is carried out. Taking the model data under different pedestrian flow volume scenarios as the basic sample data, the weights of the two evaluation indexes are determined by using Equations (30)–(35) in Section 3.2.3 of the entropy weight method model, and the final evaluation model is shown in Equation (37).

(37) $$Ys=0.5512\times ps+0.4488\times \bar{I}$$

### 4.3. Analysis of Safety Evaluation Results

**Figure 10:**
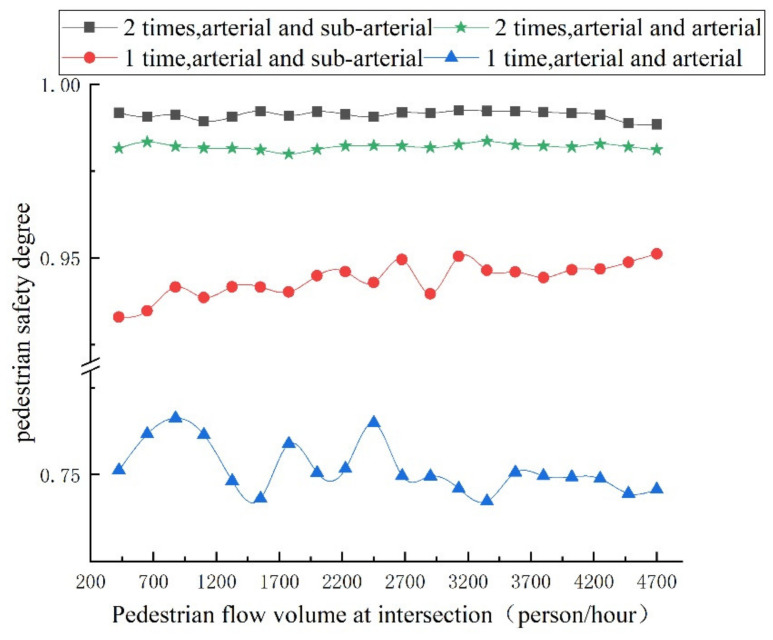
Variation trend of pedestrian crossing safety with different pedestrian flow volumes.

**Figure 11:**
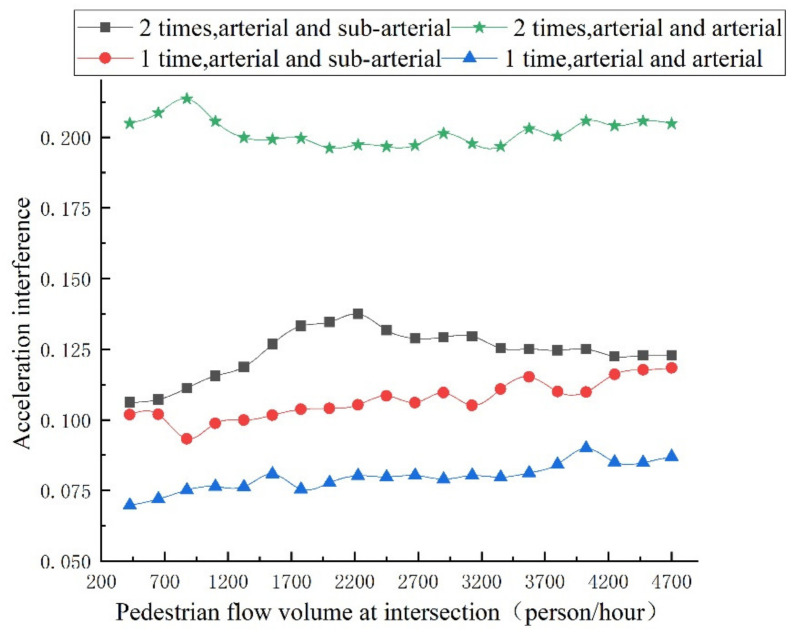
Trend of vehicle acceleration interferes with different pedestrian flow volumes.

**Figure 12:**
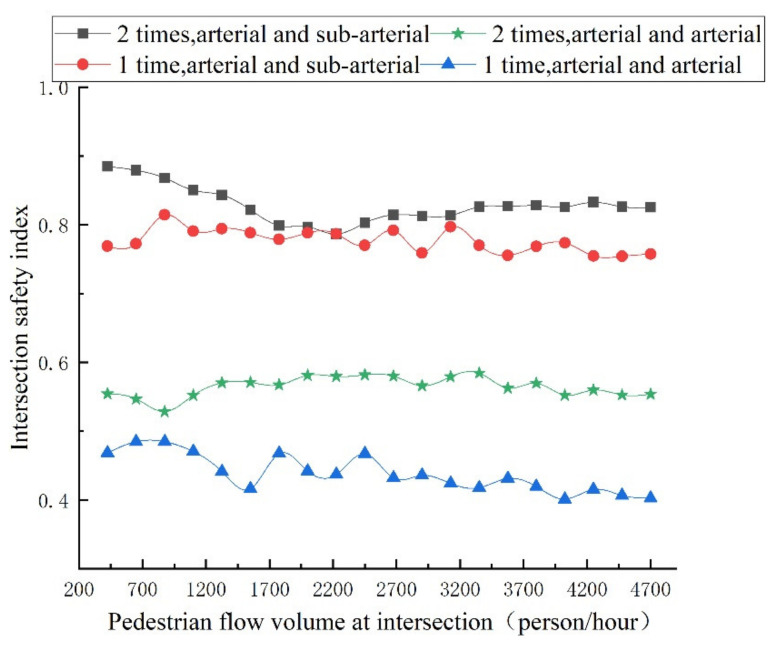
Trend of intersection safety index with different pedestrian flow volumes.

Under the influence of various social forces, the changing trend of the accompanying pedestrian flow volume, safety degree of pedestrian crossing, and vehicle acceleration interference value of each evaluation index are shown in Figure 10 and Figure 11, respectively, and the intersection safety index is shown in Figure 12.

It can be seen from Figure 10 that the safety degree of pedestrian crossing and safety degree of pedestrian crossing in the two-times crossing of pedestrians is significantly higher than that in the one-time crossing, and the safety degree of the pedestrian crossing at the intersection of arterial and arterial in the one-time crossing is relatively low. Due to the consideration of the social force of conformity, the increase in pedestrian flow volume will improve the safety perception of pedestrians at the intersection at a low level. With the increase in pedestrian flow volume, the change in 1-time crossing safety degree is more obvious than two-times crossing. In the case of one-time crossing between arterial and arterial, when the pedestrian flow volume of intersection is less than 2700 (person/hour), it is greatly affected by pedestrians, and when the pedestrian flow volume of intersection is more than 2700 (person/hour), the safety degree of pedestrian crossing basically remains around 0.75. The pedestrian safety of the intersection with one-time crossing between arterial and sub-arterial increases, because there are fewer conflicts between people and cars at this intersection, and the distance of the sub-arterial road is shorter than that of the arterial road; therefore, the pedestrian perception is safer.

It can be seen from Figure 11 that for the acceleration interference value for the vehicles at the intersection, the acceleration interference index results of the intersection will be greatly different when the pedestrian flow volume is low. The reason is that the pedestrian interference is small when pedestrian volume is low. With the increase in pedestrian flow volume at the intersection, the acceleration interference value of vehicles at the intersection with 1-time crossing between arterial and arterial is relatively stable, and the conflict between pedestrian and vehicles is completely separated. Only when the pedestrian flow volume is low (less than 700 person/hour) does the acceleration interference value increase slightly. When the intersection with two-times crossing between arterial and sub-arterial pedestrian flow volume is less than 2200 (person/hour), the increase in pedestrians has a significant influence on the acceleration of vehicles. After more than 2200 (person/hour), the increase in pedestrians has little impact on vehicle acceleration. For the one-time crossing of pedestrians, the increase in pedestrian flow volume at the intersection makes the acceleration interference value continuously increase.

The entropy weight method is used to fuse the two indicators of pedestrian safety and vehicle acceleration interference at the intersection, and the intersection safety index is obtained, as shown in Figure 12. The change in pedestrian flow volume has different effects on the safety of different types of intersections. The safety index of arterial and sub- arterial intersection scenes is significantly higher than that of arterial and arterial intersection scenes, and the two-times crossing scenes are higher than that of one-time crossing scenes.

In the arterial and sub-arterial intersection scene, when the pedestrian flow volume at the intersection is less than 2200 (person/hour), the safety index trend of two-times crossing is opposite to that of the one-time crossing; at 2200 (person/hour), the safety index of the two scenarios is close.

For the one-time pedestrian crossing scene, when the pedestrian flow volume at the intersection is less than 875 (person/hour), the safety index of the intersection increases with the increase in pedestrians flow volume. It indicates that in the one-time crossing scene, people with a companion will improve the safety perception of pedestrians, thus improving the safety index of the intersection. When the pedestrian flow volume is greater than 875 (people/hour), the intersection safety index fluctuates, but the overall trend is that the safety degree decreases.

For the two-times pedestrian crossing scene, the trend of the intersection safety index is firstly decreased, then slightly increased and finally maintained at a relatively stable value. Therefore, in practice, corresponding safety protection measures should be set according to the characteristics of pedestrian flow volume in different scenarios. Only relying on the yield pedestrian rule will not improve the perceived safety of pedestrians at intersections. At this time, the right turn red light time should be set or the intersection phase timing should be optimized to reduce conflicts, improve pedestrian crossing perceived safety, and better improve the intersection safety level.

**Figure 13:**
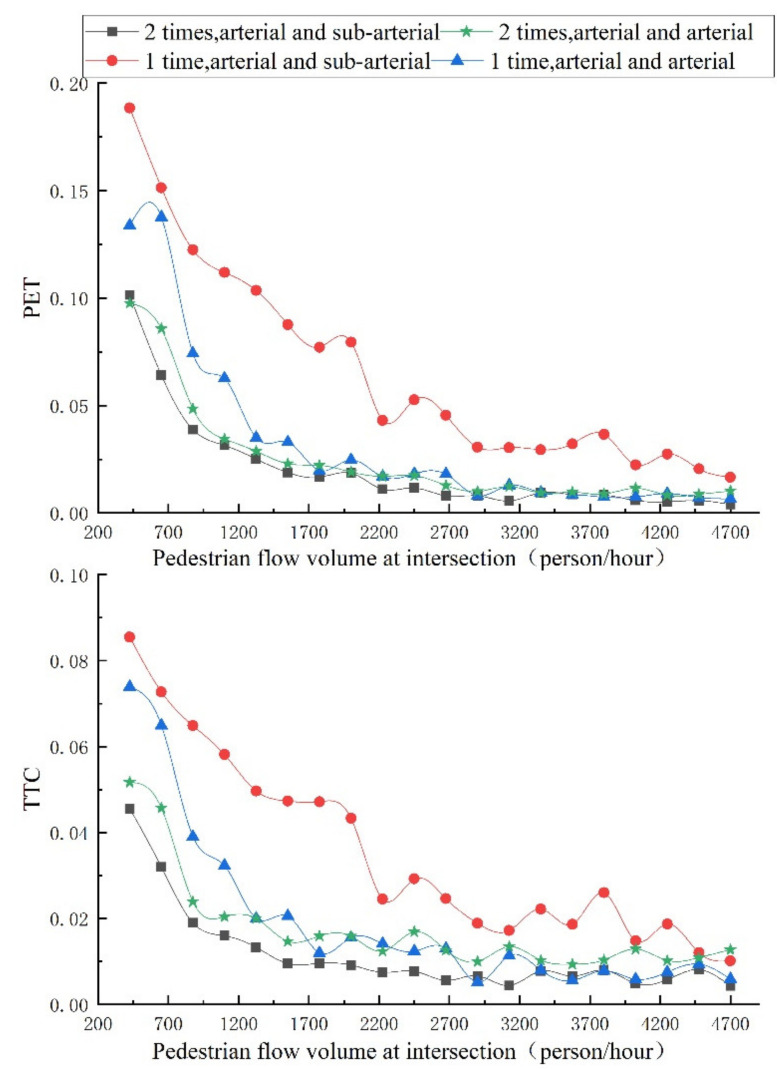
Comparison of average post-encroachment time (PET) and time-to-collision (TTC) indicators with different pedestrian flow volumes.

SSAM safety analysis software is used to investigate the variation trend of post-encroachment time (PET) and time-to-collision time (TTC), two indicators of intersection, as shown in Figure 13.

It can be analyzed from Figure 13 that from the two indicators, PET and TTC, the increase in pedestrian flow will cause the intersection safety index to change significantly, and there is little difference in the overall change trend of the two indicators.

In the intersection between two arterial, and intersection between arterial and sub-arterial t, the safety of the one-time crossing of pedestrian is significantly higher than that of the two-time crossing of pedestrian. This evaluation result clearly does not take into account the improvement in the central safety island to the pedestrian safety at the intersection. By comparing the comprehensive safety evaluation index with the results of the TTC and PET index evaluation (Figure 13), it can be found that the safety of two-times crossing scene is higher than that of the one-time crossing scene after the evaluation using the social force model.

Since post-encroachment time (PET) and time-to-collision (TTC) are both severity indicators to describe the conflict, considering the pedestrian yield rule, it is not enough to consider only the spatial location relationship. Therefore, it is necessary to simultaneously consider the pedestrian perception characteristics and conduct a comprehensive evaluation of the intersection in combination with factors such as signal lamp group and pedestrian crossing psychology. Therefore, the results of the evaluation method proposed in this study are more practical and also meet the safety evaluation needs of intersections at the present stage.

Table 5 shows the standard deviation of relevant indicators, which reflects the fluctuation of the influence of pedestrian volume at each intersection on the intersection safety indicators. Regarding pedestrian safety, the index of the two-times crossing scene fluctuates less; regarding vehicle acceleration interference, the arterial and arterial intersection scene has a minor fluctuation, and the actual situation is also the same. The 2-times crossing has a higher guarantee for pedestrian safety, and the phase configuration of the arterial intersection scene is more conducive to vehicle dissipation. Therefore, it can be postulated that the intersection safety index obtained by integrating the driver-vehicle indicators with the entropy weight method can better integrate the characteristics of the driver-vehicle indicators. The standard deviations of post-encroachment time PET and time-to-collision TTC are both larger in 1-time crossing of pedestrians, indicating that the number of accompanying people significantly increases the pedestrian–vehicle conflict at one-time crossing of pedestrian.

## 5. Conclusions

Based on the above cases, we can draw the following conclusions:

(1) Oriented to different characteristics of channelization, facility conditions, traffic composition, and flow volume characteristics of intersections in arteries, the social force model can describe the perceived safety of pedestrians and vehicles in the process of crossing the street.
(2) Different safety evaluation indicators lead to different results; however, the social force model considers a variety of influencing factors of street crossing, and its associated safety indicators can describe the safety perception of relevant participants considering pedestrian yield rules. Moreover, the entropy weight method can be used to comprehensively evaluate the safety of intersections.
(3) Currently, with the implementation of the pedestrian yield rule, the safety of pedestrian crossing at intersections across different arterials has been greatly improved. In this scenario, greater attention should be given to the pedestrian crossing safety perception level.
(4) From the case study, we can conclude that the safety level of an intersection changes with the fluctuation of pedestrian flow volume; moreover, different types of intersections in arterials have different sensitivities to changes in intersection safety with the fluctuation of pedestrian flow volume. The safety index between the arterial and sub-arterial intersection scenarios is significantly higher than that between the arterial and arterial intersection scenarios, and the safety index of intersections with two times crossing of pedestrians is significantly higher than that of intersections with one time crossing of pedestrians.

In the scenario of one-time crossing, for pedestrians, being with or without a companion is a key factor to their safety. Therefore, in practice, the choice of pedestrian yield or signal control can be further comprehensively decided by considering factors such as different people–vehicle flow volume, geometric dimensioning of the intersection, one-time or two-time pedestrian crossings, and the configuration of the signal lamp group.

There are two main contributions of this study. The first proposes the demand for pedestrian and driver crossing safety under the yield rule and applies the social force model to the intersection safety evaluation. The second contribution is integrating the crossing safety of pedestrians and drivers, improving the existing traffic safety evaluation methods and optimizing the implementation scene of pedestrian yield rules, and providing a certain theoretical basis for the design of intersection signal controls in the future.

However, there are some limitations that still require further consideration for future research. Although many factors such as signal lights, zebra crossing, and pedestrian crossing psychology are considered in the study, in a follow-up study, other influencing factors, such as other road users and the channelization of the intersection should be analyzed in depth. In addition, this paper mainly focused on the safety of vehicles and individuals at the intersection; however, the efficiency of pedestrian yield at intersections in arterials is also crucial, which is an issue that we will study in the future. Moreover, future work will investigate a comprehensive evaluation of safety and efficiency.

Intersections are the gathering place of urban pedestrians and vehicle flow. Intersection safety is closely related to people’s health, especially for children and the elderly. The conclusions of this research can help improve intersection safety, greatly improve people’s perception of safety when crossing the street, and promote the health and well-being of residents.

## Figures and Tables

**Table 1:** Signal phase and pedestrian crossing pattern of intersections surveyed.

	Hechuan Road (Arterial) Wuzhong Road (Arterial) Intersection	Zhayin Road (Arterial) Yinxing Road (Arterial) Intersection	Nenjiang Road (Arterial) Zhongyuan Road (Sub-Arterial) Intersection	Zhoujiazui Road (Arterial) Longchang Road (Sub-Arterial) Intersection
Grade of intersection	Arterial and arterial	Arterial and arterial	Arterial and sub-arterial	Arterial and sub-arterial
Pedestrian crossing form	1-time crossing	East entrance, 1-time crossing. Other entrance, 2-times crossing	1-time crossing	Arterial road with 2-times crossing and sub-arterial road with 1-time crossing
Number of signal lamp phases	4	3	3	5
Intersection type	1-time, arterial and arterial	2-times, arterial and arterial	1-time, arterial and sub-arterial	2-times, arterial and sub-arterial
Conflict type	People–vehicle	Pedestrian–vehicle Vehicle–vehicle	Pedestrian–vehicle Vehicle–vehicle	People–vehicle

**Table 2:** Parameter values in Vissim simulation.

Vissim Simulation Parameter	Values
Front minimum distance	0.5 m
Added value of safety distance	2
Multiple of safety distance	3
Visual distance	60 m
Safety distance reduction factor	0.6

**Table 3:** Values of measurable parameters in the social force model.

Measurable Parameters	Values
Pedestrian expected passing speed	4.32 km/h
Vehicle expected passing speed	15 km/h
Average pedestrian mass	65 kg
Average vehicle mass	1506 kg (small); 18,000 kg (large)
Visual angle	120°
Offline pedestrian reduction factor	0.2

**Table 4:** Estimated values of non-measurable parameters.

Parameters	Values	Parameters to Describe
$A_{cc}$	214.00	Interaction strength coefficient between vehicles
$B_{cc}$	111.60	Influence range coefficient between vehicles
$A_{pp}$	25.72	Interaction strength coefficient between people
$B_{pp}$	2.06	Influence range coefficient between people
$A_{p}$	57.84	Strength coefficient of attraction between people
$A_{\alpha B}$	53.36	Interaction strength coefficient between human and boundary
$B_{\alpha B}$	47.50	Influence range coefficient of people and boundary
$A_{D}^{att}$	80.98	Attraction coefficient of signal lamp
$B_{{}_{D}}^{att}$	61.42	Attraction range coefficient of signal lamp
$A_{cp}$	50.42	Interaction strength coefficient of person on vehicle
$B_{cp}$	60.87	Influence range coefficient of person on vehicle
$A_{pc}$	87.05	Interaction strength coefficient of vehicle to human
$B_{pc}$	64.31	Influence range coefficient of vehicle on people
$A_{D}$	13.82	Repulsion coefficient of signal lamp
$B_{D}$	4.66	Repulsion range coefficient of signal lamp

**Table 5:** Standard deviation of each index.

Intersection Type	Pedestrian Safety	Vehicle Acceleration Interference	Intersection Safety Index	Time-To-Collision	Post-Encroachment Time
1-time crossing, arterial and arterial	0.0069	0.0048	0.0262	0.0189	0.0388
2-times crossing, arterial and arterial	0.0008	0.0046	0.0143	0.0111	0.0245
1-time crossing, arterial and sub-arterial	0.0048	0.0065	0.0163	0.0214	0.0472
2-times crossing, arterial and sub-arterial	0.0011	0.0084	0.0256	0.01	0.0234

## Data Availability

The data that support the findings of this study are available from the corresponding author, upon reasonable request.
